# Age-related changes in clinical parameters and their associations with common complex diseases

**DOI:** 10.3892/br.2015.505

**Published:** 2015-08-05

**Authors:** YOSHIKO MURAKATA, TETSUO FUJIMAKI, YOSHIJI YAMADA

**Affiliations:** 1 Department of Human Functional Genomics, Life Science Research Center, Mie University, Tsu, Mie 514-8507, Japan; 2 Department of Medical Genomics and Proteomics, Institute of Basic Sciences, Graduate School of Medicine, Mie University, Tsu, Mie 514-8507, Japan; 3 Department of Cardiovascular Medicine, Inabe General Hospital, Inabe, Mie 511-0428, Japan; 4 Core Research for Evolutionary Science and Technology (CREST), Japan Science and Technology Agency, Tokyo 102-0076, Japan

**Keywords:** aging, hypertension, type 2 diabetes mellitus, dyslipidemia, chronic kidney disease

## Abstract

The aim of the present study was to clarify the age-related changes in 13 clinical parameters and their associations with common complex diseases. Study subjects comprised 6,027 community-dwelling individuals who were recruited to a population-based longitudinal genetic epidemiological study. Bonferroni's correction was applied to compensate for multiple comparisons of association and P<0.0011 was considered statistically significant. Body mass index and waist circumference increased with age up to ~50 years and decreased thereafter in men, whereas the two parameters increased linearly with age in women. The prevalence of obesity was highest (41.1%) in men aged 40–49 years, after which it decreased with age. The prevalence of obesity in women increased with age to ≤32.2% in those aged ≥70 years. Systolic and mean blood pressure (BP), as well as pulse pressure, increased linearly with age in all subjects, whereas diastolic BP increased with age up to ~60 years and subsequently decreased. The prevalence of hypertension increased with age to ≤69.9 or 68.5% at age ≥70 years in men and women, respectively. The fasting plasma glucose level, blood hemoglobin A_1c_ content and the prevalence of type 2 diabetes mellitus increased gradually with age in men and women. The serum triglyceride concentration increased with age up to ~50 years and decreased thereafter in men, whereas it increased linearly with age in women. The prevalence of hypertriglyceridemia increased to a peak of 56.8% at age 50–59 years and subsequently decreased in men, whereas in women it increased with age to ≤34.9% at ≥70 years. The serum high-density lipoprotein (HDL)-cholesterol concentration increased with age up to ~50 years and decreased thereafter in women. The prevalence of hypo-HDL-cholesterolemia increased gradually with age in women. The serum concentration of low-density lipoprotein (LDL)-cholesterol increased with age up to ~50 years and subsequently declined in men, whereas it increased linearly with age in women. The prevalence of hyper-LDL-cholesterolemia increased with age to ≤53.4% at 50–59 years in men and ≤63.9% at 60–69 years in women and it decreased thereafter in the two genders. The serum creatinine concentration and the estimated glomerular filtration rate increased or decreased linearly with age, respectively. The prevalence of chronic kidney disease (CKD) increased with age to ≤45.1 or 39.6% at ≥70 years in men and women, respectively. Therefore, these results indicate that 13 clinical parameters, as well as the prevalence of obesity, hypertension, type 2 diabetes mellitus, dyslipidemia and CKD, were significantly associated with age. They may therefore prove informative for the prevention of these diseases and contribute to the achievement of a healthy long life and successful aging.

## Introduction

Aging is an inevitable, complex and multifactorial process that is characterized by the progressive degeneration of tissues and organ systems in humans (1). Although the aging process is largely determined by genetics, it is influenced by various environmental factors, including diet, exercise, smoking, alcohol intake and exposure to microorganisms, pollutants and ionizing radiation (1). People of the same age may thus differ markedly in terms of their physical appearance and physiological status (1). In addition, in the majority of developed countries, women typically outlive men by 7–10 years (1,2). Recent research has also shown that childhood personality and education, as well as behavioral factors, contribute to longevity (3).

Physiological changes associated with aging lead to a decrease in the function of various organ systems (4,5). Given that aging also increases mortality risk as a function of time, it is important to understand precisely the anatomic and physiological changes attributed to the normal aging process. A key medical and social aim is to extend the healthy life span of humans and to prevent prolonged immobility or hospitalization of the elderly due to serious conditions, such as cardiovascular disease, stroke or fractures.

The present study examined age-related physiological changes in 13 clinical parameters and their associations with obesity, hypertension, type 2 diabetes mellitus, dyslipidemia, and chronic kidney disease (CKD) in community-dwelling Japanese individuals. The aim of the study was to contribute to the prevention of these diseases and to promote the achievement of a healthy long life and successful aging in the elderly.

## Subjects and methods

### 

#### Study population

Study subjects comprised 6,027 community-dwelling individuals who were recruited to a population-based cohort study (Inabe Health and Longevity Study) in Inabe City (Mie, Japan). The Inabe Health and Longevity Study is a longitudinal genetic epidemiological study of atherosclerotic, cardiovascular and metabolic diseases (6–9). Detailed methods for the recruitment of the study subjects and for the collection and storage of medical examination data and genomic DNA samples, as well as characteristics of the study subjects (3,352 men, 2,675 women) with regard to all measurements in a 5-year follow-up, were as described previously (6).

#### Examination of age-related changes in clinical parameters and the prevalence of common complex diseases

Age-related changes in a total of 13 clinical parameters, including body mass index (BMI); waist circumference; systolic, diastolic and mean blood pressure (BP); pulse pressure; fasting plasma glucose concentration; blood glycosylated hemoglobin (hemoglobin A_1c_) content; serum concentrations of triglycerides, high-density lipoprotein (HDL)-cholesterol and low-density lipoprotein (LDL)-cholesterol; serum creatinine level; and estimated glomerular filtration rate (eGFR), were examined. Age-related changes in the prevalence of obesity, hypertension, type 2 diabetes mellitus, hypertriglyceridemia, hypo-HDL-cholesterolemia, hyper-LDL-cholesterolemia and CKD were also evaluated. Diagnostic criteria for these diseases were as follows: Subjects with obesity had a BMI of ≥25 kg/m^2^, based on the BMI criteria of obesity for Japanese and Asian populations (10), and control individuals had a BMI of <25 kg/m^2^. Subjects with hypertension either had a systolic BP of ≥140 mmHg or diastolic BP of ≥90 mmHg (or both) or had been prescribed antihypertensive medication, and control individuals had a systolic BP of <140 mmHg and diastolic BP of <90 mmHg, as well as no history of hypertension or of taking antihypertensive medication. Subjects with type 2 diabetes mellitus either had a fasting plasma glucose level of ≥126 mg/dl or blood hemoglobin A_1c_ content of ≥6.5% (or both) or had been prescribed antidiabetes medication, and control individuals had a fasting plasma glucose level of <126 mg/dl and blood hemoglobin A_1c_ of <6.5%, as well as no history of type 2 diabetes mellitus or of taking antidiabetes medication. Subjects with hypertriglyceridemia either had a serum triglyceride concentration of ≥150 mg/dl or had been prescribed antidyslipidemic medication for hypertriglyceridemia, and control individuals had a serum triglyceride level of <150 mg/dl, as well as no history of hypertriglyceridemia or of taking antidyslipidemic medication. Subjects with hypo-HDL-cholesterolemia had a serum HDL-cholesterol concentration of <40 mg/dl or had been prescribed antidyslipidemic medication for hypo-HDL-cholesterolemia, and control individuals had serum HDL-cholesterol of ≥40 mg/dl, as well as no history of hypo-HDL-cholesterolemia or of taking antidyslipidemic medication. Subjects with hyper-LDL-cholesterolemia had a serum LDL-cholesterol level of ≥140 mg/dl or had been prescribed antidyslipidemic medication for hyper-LDL-cholesterolemia, and control individuals had serum LDL-cholesterol of <140 mg/dl, as well as no history of hyper-LDL-cholesterolemia or of taking antidyslipidemic medication. The eGFR was calculated with the use of the simplified prediction equation derived from that in the Modification of Diet in Renal Disease Study and proposed by the Japanese Society of Nephrology: eGFR (ml min^−1^ 1.73 m^−2^) = 194 × [age (years)]^−0.287^ × [serum creatinine (mg/dl)]^−1.094^ (x 0.739 if female) (11). The National Kidney Foundation-Kidney Disease Outcomes Quality Initiative guidelines recommend a diagnosis of CKD if eGFR is <60 ml min^−1^ 1.73 m^−2^ (12). Therefore, the present study adopted the criterion of an eGFR of <60 ml min^−1^ 1.73 m^−2^ for diagnosis of CKD, and control individuals had an eGFR of ≥60 ml min^−1^ 1.73 m^−2^ and did not have a history of renal disease.

The study protocol complied with the Declaration of Helsinki and was approved by the Committees on the Ethics of Human Research of Mie University Graduate School of Medicine and Inabe General Hospital. Written informed consent was obtained from all the subjects.

#### Statistical analysis

Categorical data according to age were compared between cases and controls by the χ^2^ test. Correlations of quantitative data (clinical parameters) with age were examined by simple regression analysis with fitting to a straight line or quadratic curve. Bonferroni's correction was applied to compensate for multiple comparisons and P<0.0011 (0.05/44) was considered to indicate a statistically significant difference. Statistical tests were performed with JMP 5.1 software (SAS Institute Inc., Cary, NC, USA).

## Results

### 

#### Age-related changes in BMI and waist circumference

**Figure 1.**
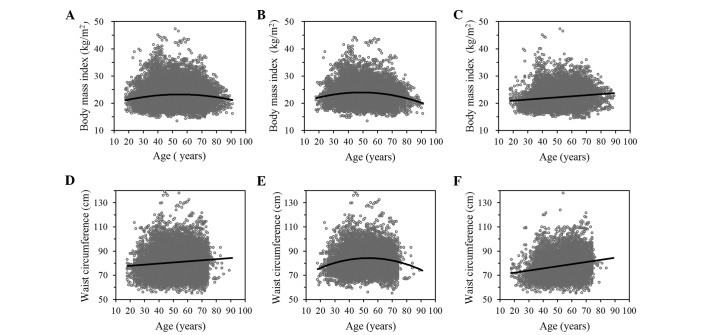
Correlation of body mass index (BMI) or waist circumference with age. Correlations were examined in longitudinal data for BMI in (A) all the subjects (27,921 measurements), (B) men (15,548) or (C) women (12,373), as well as for waist circumference in (D) all subjects (21,358), (E) men (11,817) or (F) women (9,541). The line in each panel represents a least-squares plot of the data. (A) P=6.51×10^−42^, *R*^2^=0.0074, BMI (kg/m^2^)=22.8255+0.0074*x*−0.0016 (*x*−52.4897)^2^; (B) P=8.96×10^−56^, *R^2^*=0.0174, BMI (kg/m^2^)=24.7967–0.0165*x*−0.0022 (*x*−52.4919)^2^; (C) P=8.21×10^−61^, *R^2^*=0.0216, BMI (kg/m^2^)=20.0823+0.0406*x*; (D) P=4.34×10^−60^, *R^2^*=0.0124, waist circumference (cm)=76.0696+0.0893*x*; (E) P=3.08×10^−41^, *R^2^*=0.0166, waist circumference (cm)=83.4948+0.0132*x*−0.0072 (*x*−52.4919)^2^; (F) P=1.15×10^−103^, *R^2^*=0.0478, waist circumference (cm)=68.4565+0.1776*x*. *x*, age (years).

**Figure 2.**
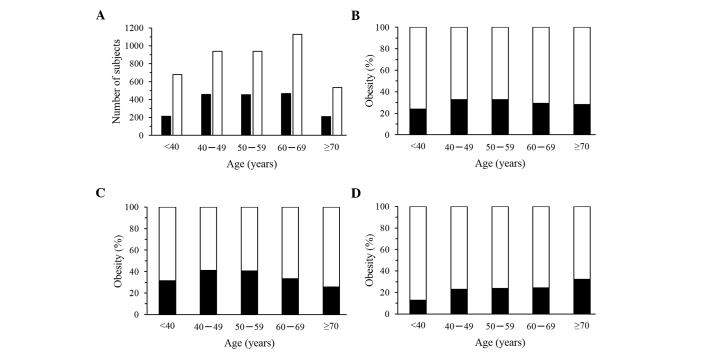
Association of the prevalence of obesity to age in cross-sectional analysis. The association of (A) the number or (B–D) percentage of subjects with obesity to age was examined for (A and B) all subjects (1,805 with obesity, 4,222 controls), as well as for (C) men (1,185 with obesity, 2,167 controls) and for (D) women (620 with obesity, 2,055 controls) separately. Subjects with obesity and the controls are represented by closed and open columns, respectively. (A and B) P=2.87×10^−4^; (C) P=8.53×10^−9^; (D) P=3.63×10^−7^.

BMI (Fig. 1A–C) and waist circumference (Fig. 1D–F) were significantly (P<0.0011) correlated with age in the longitudinal data analysis. BMI and waist circumference increased with age up to ~50 years and subsequently declined in men (Fig. 1B and E), whereas the two parameters increased linearly with age in women (Fig. 1C and F). The *R*^2^ values for BMI and waist circumference were greater in women (0.0216 and 0.0478, respectively) compared with men (0.0174 and 0.0166, respectively). The prevalence of obesity was significantly associated with age in the cross-sectional analysis performed in March 2014 (Fig. 2). The highest prevalence (41.1%) of obesity was observed in men aged 40–49 years, with the prevalence in men subsequently decreasing with age (Fig. 2C). In women, the prevalence of obesity increased gradually with age, reaching a value of 32.2% in those aged ≥70 years (Fig. 2D).

#### Age-related changes in BP and pulse pressure

**Figure 3.**
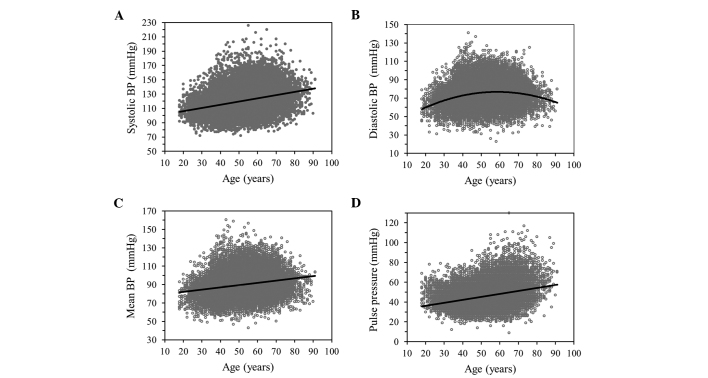
Correlation of systolic blood pressure (BP), diastolic BP, mean BP or pulse pressure with age. Correlations were examined for (A) systolic BP, (B) diastolic BP, (C) mean BP or (D) pulse pressure in longitudinal data for all the subjects (27,911 measurements). The line in each panel represents a least-squares plot of the data. (A) P<1.00×10^−64^, *R^2^*=0.1081, systolic BP (mmHg)=97.4221+0.4443*x*; (B) P=4.75×10^−156^, *R^2^*=0.0445, diastolic BP (mmHg)=69.0468+0.1400*x*−0.0113 (*x*−52.4897)^2^; (C) P<1.00×10^−64^, *R^2^*=0.0534, mean BP (mmHg)=77.2966+0.2433*x*; (D) P<1.00×10^−64^, *R^2^*=0.1101, pulse pressure (mmHg)=30.1858+0.3015*x*. *x*, age (years).

**Figure 4.**
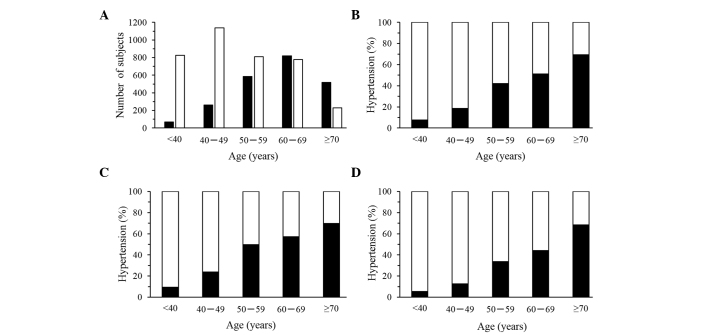
Association of the prevalence of hypertension to age in cross-sectional analysis. The association of (A) the number or (B-D) percentage of subjects with hypertension to age was examined for (A and B) all the subjects (2,250 with hypertension, 3,777 controls), as well as for (C) men (1,408 with hypertension, 1,944 controls) and for (D) women (842 with hypertension, 1,833 controls) separately. Subjects with hypertension and the controls are represented by closed and open columns, respectively. (A and B) P=1.09×10^−218^; (C) P=1.08×10^−124^; (D) P=9.53×10^−99^.

Systolic, diastolic and mean BP, as well as pulse pressure, were significantly correlated with age in the longitudinal data analysis (Fig. 3). Systolic BP (Fig. 3A), mean BP (Fig. 3C) and pulse pressure (Fig. 3D) increased linearly with age, whereas diastolic BP (Fig. 3B) increased with age up to ~60 years and decreased thereafter. The *R*^2^ values increased according to the rank order of diastolic BP (0.0445) <mean BP (0.0534) <systolic BP (0.1081) <pulse pressure (0.1101). The prevalence of hypertension was significantly associated with age in the cross-sectional analysis (Fig. 4), increasing with age to ≤69.9 or 68.5% in men and women, respectively, aged ≥70 years (Fig. 4C and D).

#### Age-related changes in fasting plasma glucose concentration and blood hemoglobin A_1c_ content

**Figure 5.**
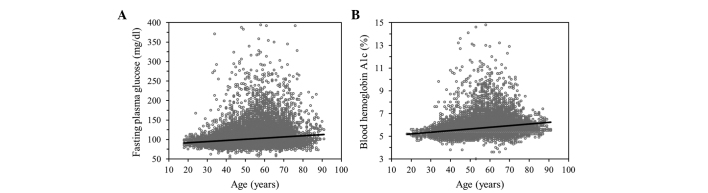
Correlation of fasting plasma glucose level or blood hemoglobin A_1c_ content with age. Correlations were examined for (A) fasting plasma glucose level (28,080 measurements) or for (B) blood hemoglobin A_1c_ (21,018) in longitudinal data for all the subjects. The line in each panel represents a least-squares plot of the data. (A) *P*=1.16×10^−181^, *R^2^*=0.0290, fasting plasma glucose (mg/dl)=86.0027+0.2916*x*; (B) *P*<1.00×10^−64^, *R^2^*=0.0687, blood hemoglobin A_1c_ (%)=4.9157+0.0146*x*. *x*, age (years).

**Figure 6.**
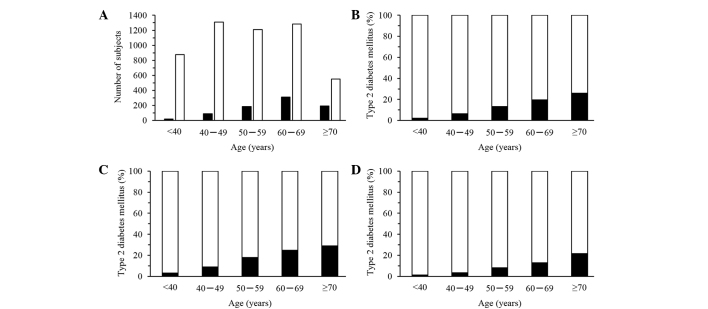
Association of the prevalence of type 2 diabetes mellitus to age in cross-sectional analysis. The association of (A) the number or (B-D) percentage of subjects with type 2 diabetes mellitus to age was examined for (A and B) all the subjects (797 with type 2 diabetes mellitus, 5,230 controls), as well as for (C) men (562 with type 2 diabetes mellitus, 2,790 controls) and for (D) women (235 with type 2 diabetes mellitus, 2,440 controls) separately. Subjects with type 2 diabetes mellitus and controls are represented by closed and open columns, respectively. (A and B) P=1.41×10^−66^; (C) P=1.70×10^−41^; (D) P=3.14×10^−26^.

The fasting plasma glucose level (Fig. 5A) and blood hemoglobin A_1c_ content (Fig. 5B) were significantly correlated with age in the longitudinal data analysis, with the two parameters gradually increasing with age (*R*^2^=0.0290 and 0.0687, respectively). The prevalence of type 2 diabetes mellitus was significantly associated with age in the cross-sectional analysis (Fig. 6), increasing with age in men (Fig. 6C) and women (Fig. 6D). The prevalence of type 2 diabetes in men was approximately twice that in women for each age group.

#### Age-related changes in serum triglyceride concentration

**Figure 7.**
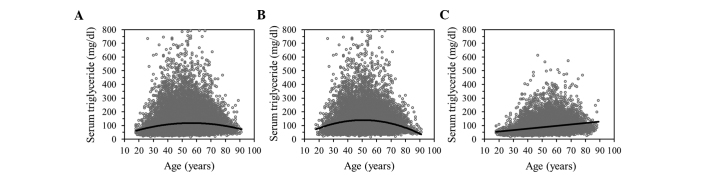
Correlation of serum triglyceride concentration with age. Correlations were examined for serum triglyceride concentration in longitudinal data for (A) all the subjects (28,040 measurements), for (B) men (15,639) and for (C) women (12,401). The line in each panel represents a least-squares plot of the data. (A) P=2.12×10^−41^, *R^2^*=0.0082, serum triglyceride (mg/dl)=101.5246+0.2975*x*−0.0379 (*x*−52.4897)^2^; (B) P=6.86×10^−49^, *R*^2^=0.0150, serum triglyceride (mg/dl)=152.3008–0.2561*x*−0.0635 (*x*−52.4919)^2^; (C) P=6.24×10^−162^, *R^2^*=0.0576, serum triglyceride (mg/dl)=34.4820+1.0486*x*. *x*, age (years).

**Figure 8.**
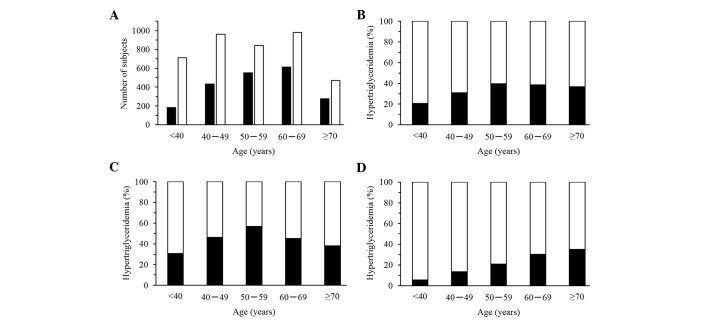
Association of the prevalence of hypertriglyceridemia to age in cross-sectional analysis. The association of (A) the number or (B-D) percentage of subjects with hypertriglyceridemia to age was examined in (A and B) all the subjects (2,058 with hypertriglyceridemia, 3,969 controls), as well as in (C) men (1,497 with hypertriglyceridemia, 1,855 controls) and (D) women (561 with hypertriglyceridemia, 2,114 controls) separately. Subjects with hypertriglyceridemia and the controls are represented by closed and open columns, respectively. (A and B) P=1.20×10^−23^; (C) P=1.52×10^−19^; (D) P=5.63×10^−31^.

The serum triglyceride concentration was significantly correlated with age in the longitudinal data analysis (Fig. 7). It increased with age up to ~50 years and decreased thereafter in men (Fig. 7B), whereas it increased linearly with age in women (Fig. 7C). The *R*^2^ value for serum triglyceride was greater in women (0.0576) compared with men (0.0150). The prevalence of hypertriglyceridemia was significantly associated with age in cross-sectional analysis (Fig. 8). In men, it increased with age to a peak of 56.8% at 50–59 years and decreased thereafter (Fig. 8C), whereas in women it increased with age to reach a value of 34.9% for those aged ≥70 years (Fig. 8D).

#### Age-related changes in serum HDL-cholesterol concentration

**Figure 9.**
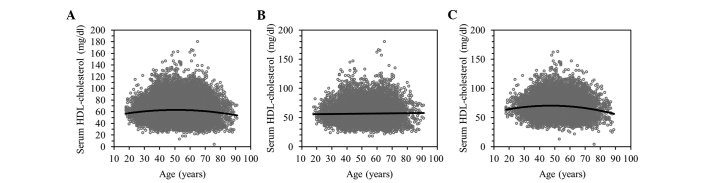
Correlation of serum high-density lipoprotein (HDL)-cholesterol concentration with age. Correlations were examined in longitudinal data for (A) all the subjects (28,005 measurements), for (B) men (15,627) and for (C) women (12,378). The line in each panel represents a least-squares plot of the data. (A) P=5.67×10^−23^, *R^2^*=0.0038, serum HDL-cholesterol (mg/dl)=64.1478–0.0213*x*−0.0058 (*x*−52.4897)^2^; (B) P=0.0136, *R^2^*=0.0004, serum HDL-cholesterol (mg/dl)=55.5027+0.0241*x*; (C) P=6.69×10^−20^, *R^2^*=0.0111, serum HDL-cholesterol (mg/dl)=74.5306–0.0840*x*−0.0080 (*x*−52.4868)^2^. *x*, age (years).

**Figure 10.**
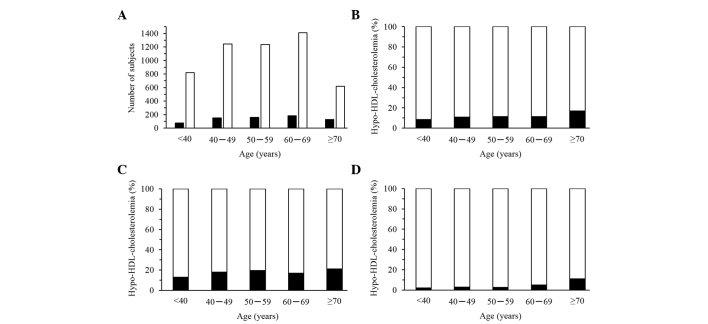
Association of the prevalence of hypo-high-density lipoprotein (HDL)-cholesterolemia to age in cross-sectional analysis. The association of (A) the number or (B-D) percentage of subjects with hypo-HDL-cholesterolemia to age was examined in (A and B) all the subjects (699 with hypo-HDL-cholesterolemia, 5,328 controls), as well as in (C) men (589 with hypo-HDL-cholesterolemia, 2,763 controls) and in (D) women (110 with hypo-HDL-cholesterolemia, 2,565 controls) separately. Subjects with hypo-HDL-cholesterolemia and the controls are represented by closed and open columns, respectively. (A and B) P=3.97×10^−6^; (C) P=0.0062; (D) P=6.46×10^−10^.

The serum concentration of HDL-cholesterol was significantly correlated with age for women, but not for men, in longitudinal data analysis (Fig. 9). Serum HDL-cholesterol increased with age up to ~50 years and decreased thereafter in women (Fig. 9C). The *R*^2^ value for serum HDL-cholesterol was greater for women (0.0111) compared with men (0.0004). The prevalence of hypo-HDL-cholesterolemia was significantly associated with age in women, but not in men, in the cross-sectional analysis (Fig. 10). In women, the prevalence of this condition increased gradually with age (Fig. 10D).

#### Age-related changes in serum LDL-cholesterol concentration

**Figure 11.**
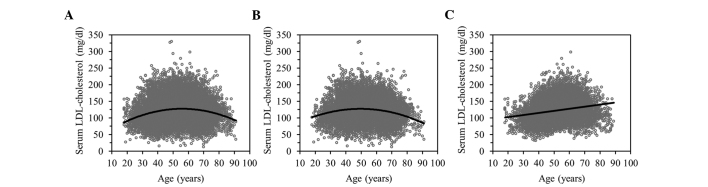
Correlation of serum low-density lipoprotein (LDL)-cholesterol concentration with age. Correlations were examined in longitudinal data for (A) all the subjects (26,833 measurements), for (B) men (14,997) and for (C) women (11,836). The line in each panel represents a least-squares plot of the data. (A) P=1.17×10^−147^, *R^2^*=0.0289, serum LDL-cholesterol (mg/dl)=117.1722+0.1907*x*−0.0288 (*x*−52.4897)^2^; (B) P=1.64×10^−66^, *R^2^*=0.0234, serum LDL-cholesterol (mg/dl)=134.9502–0.1499*x*−0.0254723 (*x*−52.4919)^2^; (C) P=1.12×10^−158^, *R^2^*=0.0591, serum LDL-cholesterol (mg/dl)=89.3721+0.6358*x*. *x*, age (years).

**Figure 12.**
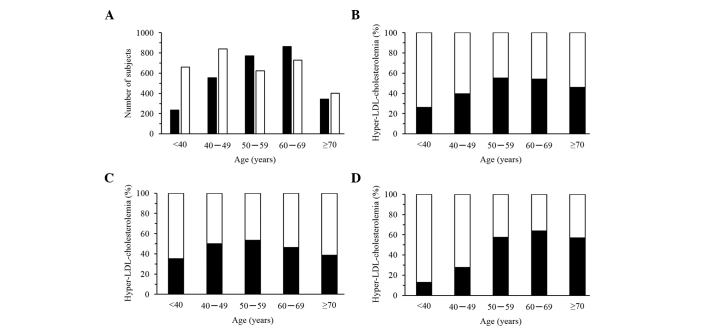
Association of the prevalence of hyper-low-density lipoprotein (LDL)-cholesterolemia to age in cross-sectional analysis. The association of (A) the number or (B-D) percentage of subjects with hyper-LDL-cholesterolemia to age was examined in (A and B) all the subjects (2,770 with hyper-LDL-cholesterolemia, 3,256 controls), as well as in (C) men (1,539 with hyper-LDL-cholesterolemia, 1,812 controls) and in (D) women (1,231 with hyper-LDL-cholesterolemia, 1,444 controls) separately. Subjects with hyper-LDL-cholesterolemia and the controls are represented by closed and open columns, respectively. (A and B) P=5.77×10^−54^; (C) P=1.71×10^−11^; (D) P=1.55×10^−32^.

The serum concentration of LDL-cholesterol was significantly correlated with age in the longitudinal data analysis (Fig. 11). It increased with age up to ~50 years and decreased thereafter in men (Fig. 11B), whereas it increased linearly with age in women (Fig. 11C). The *R*^2^ value for serum LDL-cholesterol was greater in women (0.0591) compared with men (0.0234). The prevalence of hyper-LDL-cholesterolemia was significantly associated with age in cross-sectional analysis (Fig. 12). In men, it increased with age up to a peak of 53.4% at 50–59 years and decreased thereafter (Fig. 12C), whereas in women it increased up to a peak of 63.9% at 60–69 years and subsequently declined (Fig. 12D).

#### Age-related changes in serum creatinine concentration and eGFR

**Figure 13.**
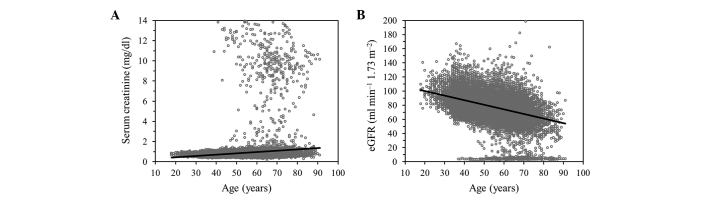
Correlation of serum creatinine concentration or estimated glomerular filtration rate (eGFR) with age. Correlations were examined for (A) serum creatinine (25,770 measurements) or (B) eGFR (25,770) in longitudinal data for all the subjects. The line in each panel represents a least-squares plot of the data. (A) P=1.62×10^−95^, *R^2^*=0.0166, serum creatinine (mg/dl)=0.1873+0.0129*x*; (B) P<1.00×10^−64^, *R^2^*=0.1769, eGFR (mg min^−1^ 1.73 m^−2^)=112.7669–0.6433*x*. *x*, age (years).

**Figure 14.**
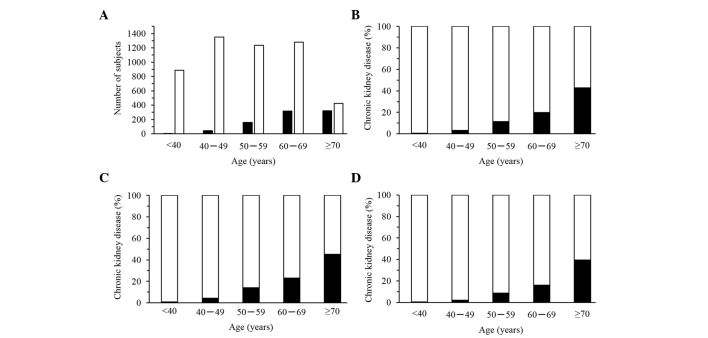
Association of the prevalence of chronic kidney disease (CKD) to age in cross-sectional analysis. The association of (A) the number or (B-D) percentage of subjects with CKD to age was examined in (A and B) all the subjects (847 with CKD, 5,180 controls), as well as in (C) men (542 with CKD, 2,810 controls) and (D) women (305 with CKD, 2,370 controls) separately. Subjects with CKD and controls are represented by closed and open columns, respectively. (A and B) P=4.14×10^−179^; (C) P=4.47×10^−104^; (D) P=9.02×10^−75^.

The serum concentration of creatinine and eGFR were significantly correlated with age in longitudinal data analysis (Fig. 13). Serum creatinine (Fig. 13A) and eGFR (Fig. 13B) increased or decreased linearly with age, respectively (*R*^2^=0.0166 and 0.1769, respectively). The prevalence of CKD was significantly associated with age in the cross-sectional analysis (Fig. 14), increasing with age to ≤45.1 or 39.6% in men and women, respectively, at ≥70 years (Fig. 14C and D).

## Discussion

The present study examined the age-related changes in 13 clinical parameters and their associations with obesity, hypertension, type 2 diabetes mellitus, dyslipidemia and CKD in community-dwelling Japanese individuals. The results indicate that these clinical parameters and disease prevalence are significantly associated with age.

Cross-sectional studies with large populations have shown that body weight and BMI increase gradually during adult life, ≤50–59 years of age, and subsequently decrease in men and women (13–15). Given the cross-sectional nature of these studies, there was a possibility of survival bias due to the higher mortality rates for obese individuals in middle adulthood (16). Longitudinal cohort studies have shown that body weight and BMI increase with age up to ~50 years in men and women, remain unchanged between 50 and 70 years in men and between 50 and 60 years in women, and subsequently decline at later ages (17–19). Waist circumference was also found to increase with age, with the extent of this increase being greater in women compared with men (20).

Aging is associated with substantial changes in body composition (21). Fat mass increases after 20–30 years of age, whereas fat-free mass (reflecting mostly skeletal muscle) progressively decreases by ≤40% from 20 to 70 years of age (22,23). Whereas fat-free mass is maximal at ~20 years of age and fat mass is maximal at 60–70 years, with the two parameters declining following these respective ages (22,23). Aging is also associated with a redistribution of body fat and fat-free mass. With aging, there is an increase in intra-abdominal fat relative to subcutaneous or total body fat, as well as a decrease in peripheral fat-free mass relative to central fat-free mass as a result of the loss of skeletal muscle (24).

The balance between energy intake and expenditure is an important determinant of body fat mass (21). Previous studies have suggested that energy intake either does not change or decreases during aging (25,26). A decrease in total energy expenditure, including that attributable to the resting metabolic rate and the thermic effect of food and physical activity, may therefore be an important factor in the gradual increase in body fat with age (27). Physical activity decreases with age (28) and this decrease has been estimated to account for about one-half of the decline in total energy expenditure that occurs with aging (27).

The present results show that BMI, waist circumference and the prevalence of obesity increased gradually with age up to ~50 years and decreased thereafter in men, whereas these parameters increased gradually with age in women. The age-related change in BMI for men was similar to that observed in previous cross-sectional and longitudinal studies (13–15,17–19). The age-related change in waist circumference for women was similar to previous observations (20), however, that of the BMI change for women was not consistent with the results of previous studies (13–15,17–19). Although the reason for this latter discrepancy is unclear, it may reflect differences in ethnicity (genetics) or environmental factors, including dietary habits, physical activity and other lifestyle aspects.

In the Framingham Heart Study, systolic BP was found to increase between the ages of 30 and ≥84 years, whereas diastolic BP increased until the fifth decade and subsequently decreased slowly from the ages of 60–84. These changes in systolic and diastolic BP resulted in an increase in pulse pressure with age (29). An increased pulse pressure due to elevated systolic BP and decreased diastolic BP in elderly individuals was shown to be an independent risk factor for cardiovascular disease (29). An increased risk of cardiovascular complications associated with an increased pulse pressure was also demonstrated in a meta-analysis including the results of several major trials (30). The Prospective Studies Collaboration examined 61 studies of BP and mortality in 1 million adults with no previous cardiovascular disease at baseline and identified that BP was strongly associated with the age-specific mortality of stroke, coronary artery disease and other vascular diseases (31).

The increase in BP with age is associated with changes in arterial and arteriolar stiffness. The stiffness of large arteries is due mostly to arteriosclerotic structural alterations and calcification, and it leads to earlier reflected pressure waves from arterioles toward the heart during BP wave propagation. These pressure waves arrive back during systole, increasing central systolic BP and widening pulse pressure (32). Large-artery stiffness and peripheral vascular resistance contribute to the increase in systolic BP with age, whereas the increase in diastolic BP at ≤50 years is mostly due to increased peripheral vascular resistance in small vessels and the subsequent decrease in diastolic BP is attributable to the increase in large-artery stiffness. Although peripheral vascular resistance may initiate hypertension, it is the acceleration of large-artery stiffness that leads to the rise in systolic BP >50 years of age (32).

The present results show that systolic and mean BP, pulse pressure and the prevalence of hypertension increased linearly with age, whereas diastolic BP increased with age up to ~60 years and decreased thereafter. The age-related changes in systolic and diastolic BP and pulse pressure in the study are thus consistent with those observed previously (29).

Aging is accompanied by an increase in glucose intolerance and the prevalence of type 2 diabetes mellitus (33,34), both of which result from an imbalance between the body's requirement for insulin (insulin sensitivity) and its ability to secrete insulin (β-cell function). Glucose intolerance in the elderly may result from impaired insulin secretion, increased peripheral insulin resistance or changes in other hormone systems. Additional factors that affect glucose tolerance in the elderly include obesity, physical inactivity, reduced dietary carbohydrate, impaired renal function and administration of certain drugs (35). The Baltimore Longitudinal Study of Aging revealed that glucose tolerance declines prominently >60 years, primarily as a result of an age-related increase in adiposity and decrease in physical activity, with the effect of age itself being moderate (36). The finding that impairment of glucose tolerance develops largely >60 years of age (36) is consistent with the results of several studies showing that insulin-mediated glucose disposal is also decreased in such elderly individuals (37–39). Loss of glucose tolerance with age is thus associated with weight gain and a sedentary lifestyle, as well as to a loss of insulin secretory function and insulin sensitivity (40).

The present results show that the fasting plasma glucose level, blood hemoglobin A_1c_ content and the prevalence of type 2 diabetes mellitus gradually increased with age in men and women. Age-related changes in these parameters in the study are thus consistent with previous observations (36–39).

Longitudinal and cross-sectional analyses of lipid profiles in a previous study with Japanese individuals showed that serum triglyceride levels increased until 50 years of age and subsequently decreased in men, whereas they increased until 70 years of age in women (41,42). An age-related change in serum HDL-cholesterol level was not observed in men, whereas this parameter decreased with age in women. In men, the serum LDL-cholesterol concentration increased until age 60 and remained unchanged from age 60–80, whereas in women it increased until age 80.

The mechanisms responsible for the development of dyslipidemia with age remain unclear, however, they may be associated with changes in the liver sinusoidal endothelium (pseudocapillarization), an increase in postprandial lipemia, insulin resistance induced by free fatty acids, growth hormone deficiency, a decline in androgen levels in men and in estrogen levels in women and a decrease in peroxisome proliferator-activated receptor α expression in the liver (43).

The present results show that the serum triglyceride level increased with age up to ~50 years and decreased thereafter in men whereas it increased linearly with age in women. The serum concentration of HDL-cholesterol did not change with age in men, whereas it increased slightly with age up to ~50 years and decreased thereafter in women. The serum LDL-cholesterol level increased with age up to ~50 years and subsequently declined in men, whereas it increased linearly with age in women. With the exception of HDL-cholesterol results for women, the age-related changes in lipid profiles in the study are thus largely consistent with those observed previously (41,42).

Renal function was previously shown to decrease gradually >30 years (44). Numerous individuals thus manifest a progressive decrease in glomerular filtration rate and renal blood flow with age, although there is wide interindividual variability. The decline in glomerular filtration rate is attributable to a reduction in the glomerular capillary plasma flow rate and the glomerular capillary ultrafiltration coefficient (44,45). Aging is also associated with altered responsiveness to vasoactive stimuli, such that responses to vasoconstrictor stimuli are enhanced whereas vasodilatory responses are impaired (45). The activities of the renin-angiotensin and nitric oxide systems also change with age (45). These physiological changes result in age-related impairment of kidney function and the development of CKD (45).

The present results show that the serum creatinine level and the prevalence of CKD increased linearly with age, whereas eGFR declined. The age-related changes in these parameters are consistent with previous observations (46,47).

There were limitations to the present study, which included: i) Substantial percentages of the subjects had undergone medical treatment for hypertension, type 2 diabetes, dyslipidemia, or CKD; ii) although the mean follow-up period for the cohort was 5 years, this time varied among individuals (from 1 to 11 years); and iii) the number of subjects differed among the different age groups.

In conclusion, the present results indicate that 13 clinical parameters, as well as the prevalence of obesity, hypertension, type 2 diabetes mellitus, dyslipidemia and CKD, are significantly associated with age. These age-related changes may have important practical implications for the clinical management of elderly individuals. Given that metabolic status is dependent on age, changes in the responses to commonly administered drugs may necessitate adjustment of dosage in the elderly. There is also a requirement for the implementation of a rational diet and exercise programs in an effort to delay or reverse some of these age-related physiological changes. These results may prove informative for the prevention of the common complex diseases examined in the present study, as well as for more serious conditions such as coronary artery disease and stroke, and they may therefore contribute to the achievement of a healthy long life and a successful aging strategy.
